# Tailoring H_2_O_2_ generation kinetics with magnesium alloys for efficient disinfection on titanium surface

**DOI:** 10.1038/s41598-020-63007-6

**Published:** 2020-04-16

**Authors:** Jimin Park, Gun Hyuk Jang, Yeon Wook Jung, Hyunseon Seo, Hyung-Seop Han, Joonho Lee, Youngmin Seo, Hojeong Jeon, Myoung-Ryul Ok, Pil-Ryung Cha, Hyun-Kwang Seok, Kwan Hyi Lee, Yu-Chan Kim

**Affiliations:** 1 Department of Materials Science and Engineering, Massachusetts Institute of Technology, Cambridge, Massachusetts, 02139 USA; 2 Center for Biomaterials, Korea Institute of Science & Technology, Seoul, 02792 Republic of Korea; 3 Division of Bio-Medical Science and Technology, KIST School, Korea University of Science and Technology, Seoul, 02792 Republic of Korea; 4 Research & Development, NuclixBio, Seoul, 08380 Republic of Korea; 5 Department of Materials Science and Engineering, Korea University, Seoul, 02481 Republic of Korea; 6 School of Advanced Materials Engineering, Kookmin University, Seoul, 02707 Republic of Korea

**Keywords:** Environmental chemistry, Biomedical engineering

## Abstract

A new antibacterial strategy for Ti has been developed without the use of any external antibacterial agents and surface treatments. By combining Mg alloys with Ti, H_2_O_2_, which is an oxidizing agent that kills bacteria, was spontaneously generated near the surface of Ti. Importantly, the H_2_O_2_ formation kinetics can be precisely controlled by tailoring the degradation rates of Mg alloys connected to Ti. Through microstructural and electrochemical modification of Mg with alloying elements (Ca, Zn), the degradation rates of Mg alloys were controlled, and the H_2_O_2_ release kinetics was accelerated when the degradation rate of Mg alloys increased. With the introduction of an *in vivo* assessment platform comprised of *Escherichia coli* (*E. coli*) and transgenic zebrafish embryos, we are able to design optimized antibacterial systems (Ti-Mg and Ti-Mg-3wt% Zn) that can selectively eradicate *E. coli* while not harming the survival rate, development, and biological functions of zebrafish embryos. We envision that our antibacterial strategy based on utilization of sacrificial Mg alloys could broaden the current palette of antibacterial platforms for metals.

## Introduction

The demand for bacterial remediation has been high in numerous fields of metal-based industries, the environment, and healthcare due to the detrimental roles of bacteria in metals, such as the formation of biofilms on metal surfaces or bacteria-induced corrosion of metals^1–4^. Thus, numerous antibacterial agents, which are generally classified into organic-based antibiotic compounds and inorganic-based metal oxides, have been applied to metals for antibacterial purposes^5–8^. Despite the breakthroughs in antibacterial agent research, the current approaches remain limited by the need to directly apply these agents onto the metal surface, unavoidably altering the intrinsic surface characteristics of metal products^9,10^. In addition, this surface treatment often results in unexpected interfacial problems, such as the desorption of organic agents or the delamination of inorganic agent layers, which can cause the uncontrolled distribution of these agents into the surrounding environment^8,11^. For example, oxidative stress exerted by delaminated antibacterial agents not only can kill bacteria but also can affect the viability and biological function of normal cells^12–14^. Therefore, an alternative method to endow metals with antibacterial functionalities beyond this conventional surface-treatment strategy has been desired.

In this work, which was inspired by traditional cathodic protection technology that utilizes electrons generated from the degradation process of Mg alloys to reduce the metal ions of a primary metal^15,16^, we applied Mg alloys to reduce O_2_ molecules near the primary metal for generating hydrogen peroxide (H_2_O_2_), which has been widely applied for antibacterial purposes^17,18^. By establishing a simple electric connection between the primary metal, such as Ti, and Mg alloys, H_2_O_2_ can be released at the surface of the primary metal according to the following electrochemical reactions^13,19^:

$$({\rm{Anode}},\,{\rm{primary}}\,{\rm{metal}}){{\rm{O}}}_{2}+2{{\rm{H}}}^{+}+2{{\rm{e}}}^{-}\to {{\rm{H}}}_{2}{{\rm{O}}}_{2}$$

$$({\rm{Cathode}},\,{\rm{Mg}}\,{\rm{alloys}})\,{\rm{Mg}}+2{{\rm{H}}}_{2}{\rm{O}}\to {\rm{Mg}}{({\rm{OH}})}_{2}+2{{\rm{H}}}^{+}+2{{\rm{e}}}^{-}$$

**Figure 1:**
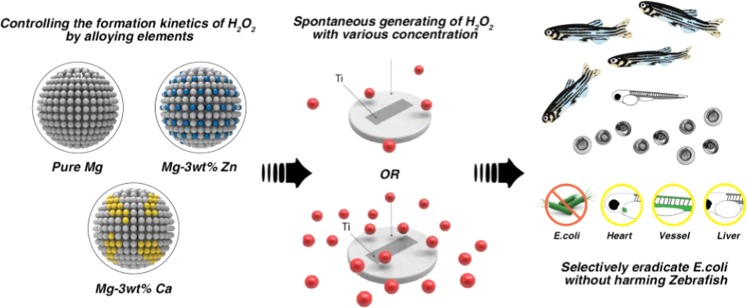
Schematic illustration of selective bacteria remediation through the biodegradability optimization of magnesium alloys. The formation kinetics of H_2_O_2_ was quantitatively controlled by systematically tailoring the microstructures and electrochemical potentials of Mg with alloying biocompatible elements (Ca and Zn). Optimized Ti-Mg alloy systems with selective bacteria remediation were discovered with an *in vivo* assessment platform comprised of *Escherichia coli* (*E. coli*) and transgenic zebrafish embryos.

The keystone of this technology is to quantitatively control the formation kinetics of H_2_O_2_ through the degradation engineering of Mg alloys. By tailoring the microstructures and electrochemical properties of Mg with secondary elements, such as Ca and Zn, we succeeded in regulating the degradation rate of Mg and H_2_O_2_ formation kinetics. In addition, to facilitate the optimization process for Mg alloys in our system, an *in vivo* assessment platform comprised of *Escherichia coli* (*E. coli*) and transgenic zebrafish embryos was constructed. By evaluating the antibacterial ability of the system using the assessment platform, we found an optimized Ti-Mg alloy system that can selectively eradicate *E. coli* without affecting the viability, development, and biological functions of zebrafish embryos (Fig. 1).

## Results and Discussions

### Effects of H_2_O_2_ on *E.coli* and Zebrafish

**Figure 2:**
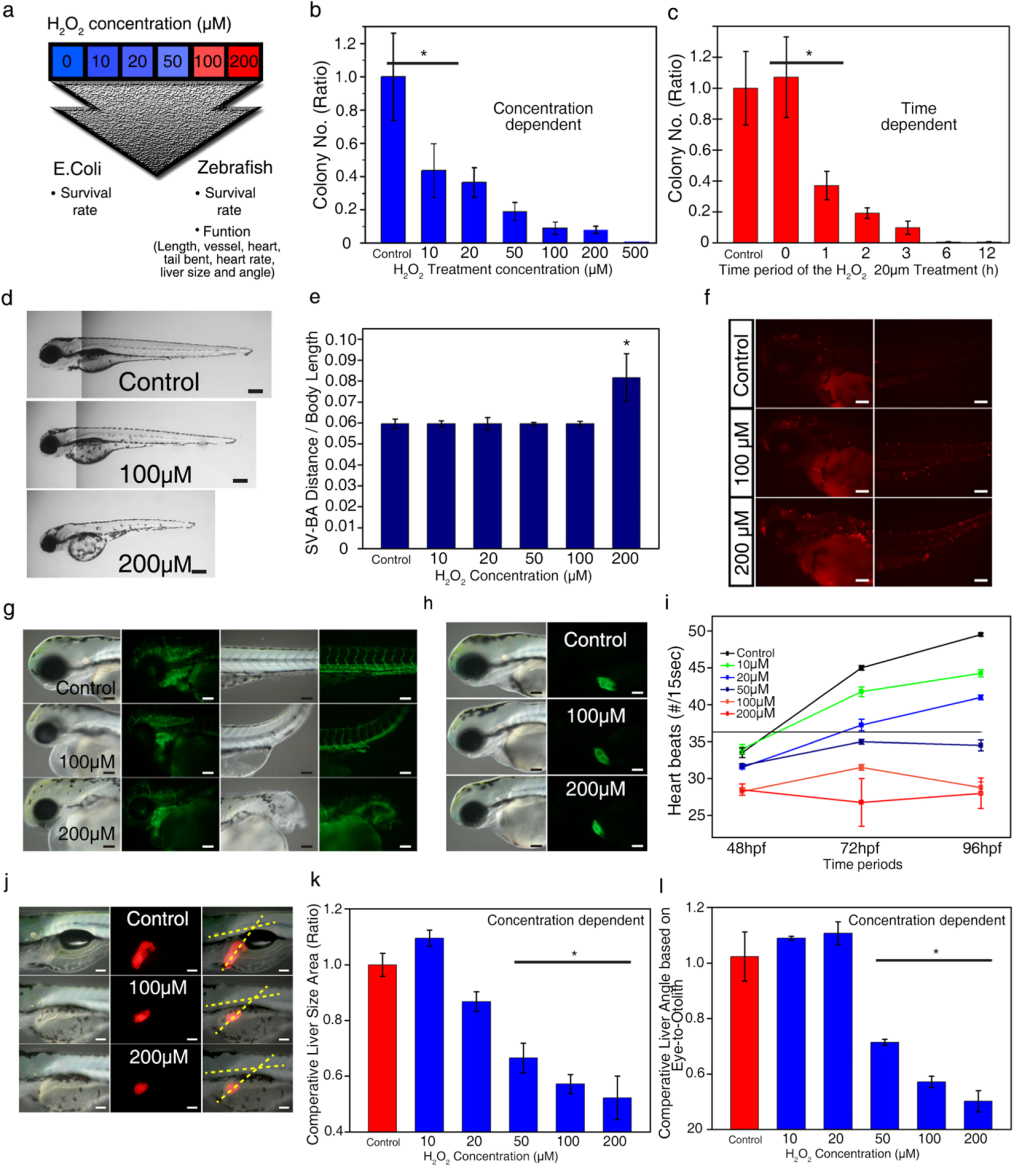
Defects of hydrogen peroxide in *E. coli* and zebrafish embryos. (**a**) Schematic describing the onset of defects due to hydrogen peroxide exposure in the *E. coli* and zebrafish embryos. (**b,c**) Concentration-dependent (**b**) and time-dependent (**c**) viability of *E. coli* treated with the H_2_O_2_ solution. (**d**) Gross morphology of the wild type zebrafish embryos treated with H_2_O_2_ at 96 hpf from a lateral view. The scale bar represents 200 µm. (**e**) Determination of the sinus venosus (SV) to bulbus arteriosus (BA) length per body length ratio at 96 hpf. (**f**) TUNEL assay of wild type zebrafish embryos at 72 hpf treated with H_2_O_2_. The scale bar represents 100 µm. (**g,h**) Optical observation of Tg(flk1:EGFP) (**g**) and Tg(cmlc2:EGFP) (**h**) zebrafish embryo phenotypes at 72 hpf upon treatment with H_2_O_2_. The scale bar represents 100 µm. (**i**) Assessment of heart functionality based on zebrafish embryo heart rates following treatment with H_2_O_2_ at 48 hpf, 72 hpf, and 96 hpf. Results from the three separate experiments are presented as heartbeat numbers compared to the control. Means ± SEM (n = 4). ∗p < 0.01 from the control group. (**j**) Tg(lfabp:DsRed) zebrafish embryo phenotype at 96 hpf upon treatment with H_2_O_2_. The angle of the developing liver was measured by ImageJ software analysis through the part shown at the dotted line. The scale bar represents 100 µm. (**k,l**) Comparative liver sizes (**k**) and angles (**l**) based on eye-to-otolith in the Tg(lfabp:DsRed) zebrafish embryos treated with H_2_O_2_. Results from the three embryo measurements are presented as liver sizes and angles compared to the control. ImageJ (version 1.52a, https://imagej.nih.gov/ij/index.html, Wayne Rasband National Institutes of Health, USA) was used for the quantification of sizes and angles of the liver.

For the systematic design of our antibacterial platform, we started by investigating the effect of H_2_O_2_ on *E. coli* and transgenic zebrafish embryos (Fig. 2a). Recently, transgenic zebrafish embryos have been utilized for initial toxicity assessment of newly developed biomaterials based on their high fecundity rates, fast development times, and low costs, and high degree of genome homology between the human and zebrafish^20–22^. Furthermore, optical transparency of zebrafish embryos enables the real-time toxicological evaluation of biomaterial^20–22^. Based on these advantages, we adopted transgenic zebrafish embryos for an initial screening of our newly developed system.

First, various concentrations of H_2_O_2_ in the range of 10 μM to 500 μM were applied to *E. coli* containing simulated sea water solutions for 2 h. The survival rates of *E. coli* gradually decreased as the concentration of H_2_O_2_ in the solutions increased (Fig. 2b), and the 50% effective concentration (*i.e*., EC_50_) values were below 10 μM. The survival rate of *E. coli* was also dependent on the H_2_O_2_ treatment period; at a fixed H_2_O_2_ concentration of 20 μM, the survival rate of *E. coli* decreased along with the H_2_O_2_ treatment period (Fig. 2c).

We then explored the effect of H_2_O_2_ on the viability and development of zebrafish embryos by applying various concentrations of the H_2_O_2_ solution for 2 h. In optical observations of gross morphology and sinus venosus (SV) to bulbus arteriosus (BA) length per body length ratio at 96 hpf^23,24^, more severe developmental disorders or delays were found with an increase in concentration of H_2_O_2_ (Fig. 2d,e). Interestingly, while the EC_50_ values against *E. coli* were below 10 μM during the 2 h of H_2_O_2_ treatment, zebrafish embryos exhibited the EC_50_ values in the range from 50 to 100 μM, indicating higher durability of zebrafish embryos against H_2_O_2_ compared to *E. coli*. In addition, no defects in hatching, deformation, and mortality were observed in zebrafish embryos treated with 20 μM H_2_O_2_ solution for 2 h (Supplementary Table 1). The developmental defects or delays in zebrafish embryos were found through optical observations at 96 hpf only when a higher concentration of H_2_O_2_ solution, greater than 50 μM, was applied, which confirmed the higher durability of zebrafish toward H_2_O_2_ compared to *E. coli*.

For a deeper understanding of the effect of H_2_O_2_ on zebrafish, we adopted transgenic zebrafish embryos, which express the green fluorescent protein (GFP) on the surface of the vasculature and cardiac tissues. Indeed, it has been known that vessel and heart development are highly correlated to the mechanisms of blood flow in the action of reactive-oxygen-species (ROS)^25–27^. A TUNEL assay (*In Situ* Cell Death Detection Kit, TMR red) was utilized to find the critical H_2_O_2_ concentration range where defects in the vessels of zebrafish embryos appeared (Fig. 2f). Scrutinized transgenic models treated with 100 and 200 μM H_2_O_2_ exhibited cardiac edema and bent tails or other defects (Fig. 2g,h), whereas the models treated with 10, 20, and 50 μM H_2_O_2_ did not show any change in phenotypes (Supplementary Fig. 1a–c). In addition, we counted heartbeats for 15 s to examine the effect of H_2_O_2_ on heart functionality at 48 hpf, 72 hpf, and 96 hpf (Fig. 2i and Supplementary Movies 1–6). The groups treated with 50 μM or more of H_2_O_2_ solutions showed deteriorated cardiac contractility in a dose-dependent manner (Fig. 2i). However, the other groups with 10 and 20 μM treatment still did not show significant defects during functional assessment (Fig. 2i and Supplementary Fig. 1d,e).

Red-fluorescent protein (RFP)-expressing transgenic zebrafish embryos were utilized to examine whether H_2_O_2_ could affect the hepatotoxicity of the embryos (Fig. 2j and Supplementary Fig. 1b). To demonstrate liver defects, the sizes and angles of the liver, the slope between the eye and otolith, and the developing liver, were assessed and compared^28^. The groups treated with 50 μM or more of the H_2_O_2_ solution exhibited significant differences in size and angle. Compared to that of the control group, the liver size and angle and the slope of the group treated with 50 μM of the H_2_O_2_ solution decreased by 67 ± 5.3% and 27.8 ± 0.27° and 7°, respectively (Fig. 2j–l and Supplementary Fig. 1b). In the groups treated with 100 and 200 μM of the H_2_O_2_ solution, the liver sizes and angles decreased by approximately 55% and 10°, respectively (Fig. 2j–l).

These comprehensive evaluations underscored the importance of controlling H_2_O_2_ treatment conditions, such as H_2_O_2_ solution concentration and incubation time, for selective bacteria remediation. Although an increase in either H_2_O_2_ concentration or incubation time resulted in a gradual decrease in the viability of *E. coli*, the excessive oxidative stress induced by the H_2_O_2_ solution with a concentration greater than 50 μM for 2 h also led to a decrease in viability, developmental delays, and defects in organ functions of zebrafish embryos. Put together, these findings suggested that applying H_2_O_2_ in the range of 20 μM to 50 μM for 2 h could be an optimized condition for the effective remediation of *E. coli*, with negligible influences on zebrafish embryos.

### Design of Mg alloy with different corrosion properties

After finding an ideal H_2_O_2_ treatment condition, we then investigated how the H_2_O_2_-releasing kinetics of the Ti-Mg system could be effectively tuned, which in turn allowed us to optimize the system’s H_2_O_2_-releasing kinetics for selective *E. coli* remediation. Considering the fact that electrons generated during the degradation process of Mg convert oxygen molecules near Ti into H_2_O_2_^13,19^, we hypothesized that the degradation rate of Mg could be highly related to the H_2_O_2_-releasing kinetics of the system. Among the diverse methods that could affect the degradation rate of Mg, we adopted alloying strategies because the microstructure and electrochemical properties of Mg, which determine its degradation rate, could be precisely tuned by additions of alloying elements^29–33^.

In this study, Ca and Zn were selected as the alloying elements for Mg based on their excellent biocompatibility^29,33^ and significantly different solubility limits in primary Mg phase (maximum solubility of Ca and Zn in Mg: 1.3 wt% and 6.2 wt%, respectively)^34^. Indeed, the solubility limit of alloying element is crucial in determining the overall microstructure of the alloy, since intermetallic phase can be formed as a secondary phase when the amount of alloying element exceeds its solubility limit. On the other hand, alloying elements can be fully dissolved in Mg phase without the formation of the intermetallic phase below its solubility limit. Therefore, if the solubility limits of two alloying elements are noticeably different, totally different microstructures can be obtained even at the identical weight percentage of alloying element^29–33^. In this regard, we set the weight percentage of two alloying elements (Ca and Zn) as 3 wt%, which is higher than the solubility limit of Ca but lower than that of Zn in Mg phase. We hypothesized that intermetallic phase could be only formed in the Mg-3wt%Ca alloy whereas all the Zn atoms are fully dissolved in Mg phase in the Mg-3wt% Zn alloy under this condition. Based on this hypothesis, three different types of Mg alloys (Mg, Mg-3wt%Ca, and Mg-3wt% Zn alloys) were fabricated.

**Figure 3:**
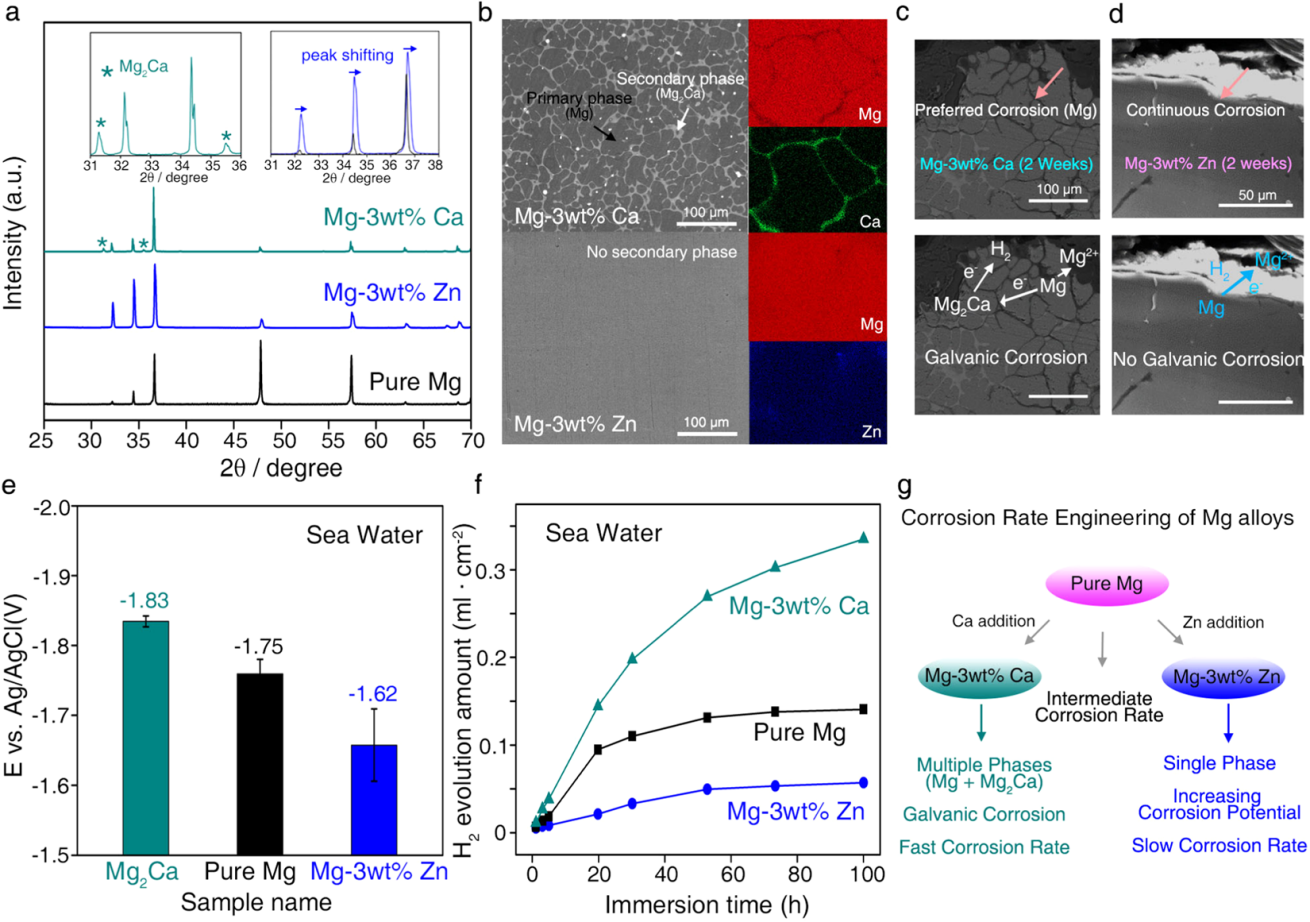
Design of Mg alloys with different degradation rates. (**a**) XRD patterns of Mg-3wt%Ca, Mg-3wt% Zn, and pure Mg. Distinct Mg_2_Ca peaks were clearly observed in Mg-3wt%Ca (a, inset, left). In the case of Mg-3wt% Zn, only a peak shift was observed without the appearance or disappearance of new peaks (a inset, right). (**b**) SEM and corresponding wavelength dispersive spectroscopy (WDS) images of Mg-3wt%Ca (top) and Mg-3wt% Zn (bottom). (**c,d**) Cross-sectional SEM images of Mg-3wt%Ca (**c**) and Mg-3wt% Zn (**d**) samples after 2 weeks of degradation. Non-uniform, galvanic corrosion between Mg and Mg_2_Ca was clearly found in Mg-3wt%Ca, whereas continuous and flat degradation was observed in Mg-3wt% Zn. (**e**) The experimental open circuit potential (OCP) values of Mg_2_Ca, pure Mg, and Mg-3wt% Zn. (**f**) Amounts of hydrogen gas evolved during degradation processes of Mg-3wt% Ca, pure Mg, and Mg-3wt% Zn alloys in simulated sea water solutions. Degradation rates increased in the series of Mg-3wt% Zn < Mg < Mg_2_Ca. (**g**) Schematics for degradation rate engineering of Mg alloys.

We first examined the microstructures of Mg, Mg-3wt%Ca, and Mg-3wt% Zn. X-ray diffraction (XRD) patterns of pure Mg and the Mg-3wt% Zn alloy showed nearly identical features, whereas new peaks from the intermetallic Mg_2_Ca phase appeared in the Mg-3wt%Ca alloy (Fig. 3a). In the case of the Mg-3wt% Zn alloy, we observed a slight Mg peak shift without any observable change in peak intensity, which indicated that the Zn elements were fully dissolved during the primary Mg phase. Scanning electron microscopy (SEM) analysis further supported the Mg-3wt%Ca alloy as consisting of a primary Mg phase and intermetallic Mg_2_Ca phases, while no distinct secondary phases existed in the Mg-3wt% Zn alloy (Fig. 3b).

Due to its different microstructure, the degradation behavior of the Mg-3wt%Ca alloy showed a distinct feature compared to Mg and the Mg-3wt% Zn alloy. Cross-sectional SEM images of degraded Mg-3wt%Ca alloys demonstrated that non-uniform degradation occurred in the Mg-3wt%Ca alloy, as the Mg_2_Ca phase was selectively degraded (Fig. 3c and Supplementary Fig. 2). This result indicated the presence of a galvanic circuit between the primary Mg and Mg_2_Ca phases, which accelerated its degradation process (Fig. 3c)^29,32^. By contrast, a uniform and flat corrosion process was observed in the case of Mg-3wt% Zn alloys, because the Zn atoms were fully dissolved into the Mg matrix without forming secondary phases (Fig. 3d).

Noticeably, we revealed that these alloying elements also affected the electrochemical properties of Mg, which is another important factor that governs its degradation rate^29–33^. The open-circuit potential (OCP), which is related to the corrosion potential of the particular Mg alloy^29^, decreased in the series of Mg-3wt% Zn > Mg > Mg-3wt% Ca (Fig. 3e). Additionally, we calculated the work function, the minimum required thermodynamic energy to extract an electron from the surface, of each metal phase. Previous reports showed that work function of metal is proportional to its corrosion potential (OCP)^29,35^. Similar to the experimental OCP trends, the theoretical work function of the Mg_2_Ca phase in Mg-3wt%Ca was smaller compared to that of Mg, whereas the addition of Zn into the Mg matrix increased the work function of the Mg matrix (Supplementary Figs. 3 and 4).

The changes in microstructure and the electrochemical properties of Mg upon the addition of alloying elements led to significant variations in the degradation rate of Mg (up to 5 fold). After 100 h of degradation in the simulated sea water solution, the amount of hydrogen gas that evolved from the Mg-3wt%Ca alloy was 0.34 ml/cm^2^_sample_, which was approximately 2.5 times higher than that produced by pure Mg (0.14 ml/cm^2^_sample_). In contrast, the Mg-3wt% Zn alloy exhibited noticeably slower degradation kinetics with significantly less evolved hydrogen gas (0.06 ml/cm^2^ samples) (Fig. 3f,g).

### Antibacterial system using bi-metal platform

**Figure 4:**
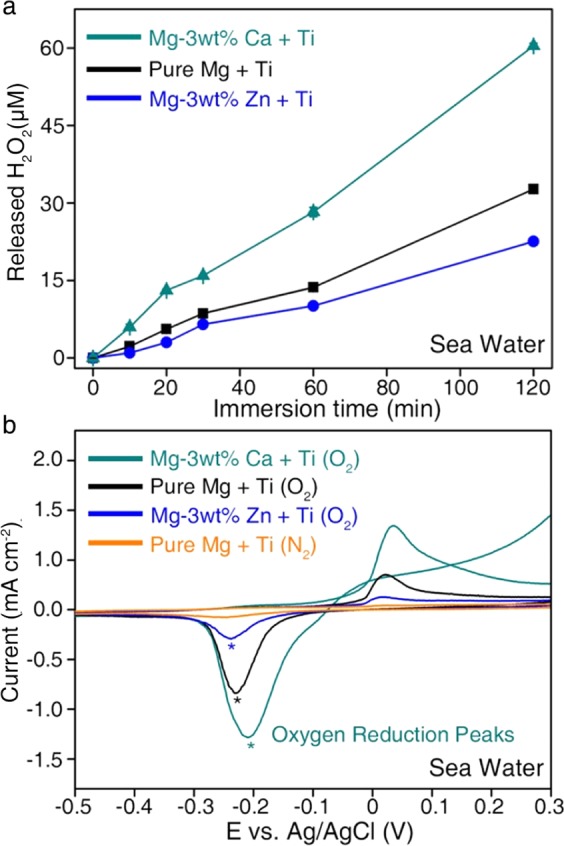
Spontaneous and tunable generation of H_2_O_2_ on integrated Ti-Mg alloy systems. (**a**) Concentration of H_2_O_2_ formed from Ti-Mg alloy systems (Ti-Mg-3wt%Ca, Ti-Mg, and Ti-Mg-3wt% Zn) in simulated sea water solutions over time. Released amounts of H_2_O_2_ in the solutions increased in the series of Ti-Mg-3wt% Zn < Ti-Mg < Ti-Mg-3wt% Ca. (**b**) Cyclic voltammetry (CV) curves for the Ti cathode connected with various Mg alloy anodes (Mg-3wt% Ca, Mg, and Mg-3wt% Zn) in O_2_-saturated sea water. As a control, the CV curve for the Ti cathode connected with the Mg anode was recorded in the N_2_-saturated solution.

To our surprise, the H_2_O_2_-releasing kinetics of the Ti-Mg alloy system showed noticeable variations depending on the type of Mg alloy (Mg, Mg-3wt%Ca, and Mg-3wt% Zn). Here, Ti and each Mg alloy (Mg, Mg-3wt%Ca, and Mg-3wt% Zn) were integrated by pouring molten Mg alloy into stainless steel mold with the Ti bar (Supplementary Fig. 5). At specific time intervals, the amount of H_2_O_2_ released from the Ti-Mg alloy system in the simulated sea water solution was measured using a fluorometric H_2_O_2_ assay kit. As shown in Fig. 3a, when we integrated Ti and pure Mg, the H_2_O_2_ concentration in the solution gradually increased over time, reaching 33 μM after 2 h of reaction. In the case of the Ti-Mg-3wt%Ca system, the H_2_O_2_-releasing kinetics were significantly accelerated and a solution of approximately 60 μM H_2_O_2_ was formed at the same reaction time. In contrast, adopting the Mg-3wt% Zn alloy in the system reduced the formation rate of H_2_O_2_, and only 23 μM H_2_O_2_ was generated after 2 h of reaction. It should be noted that the H_2_O_2_ release rate increased in the series of Ti-Mg-3wt% Zn < Ti-Mg < Ti-Mg-3wt%Ca and that this trend was identical to that of the degradation rate of Mg alloys (Figs. 3f and 4a).

Along with the fluorometric H_2_O_2_ assay results, cyclic voltammetry (CV) analyses further proved the difference in H_2_O_2_-releasing behavior depending on the type of Mg alloy connected to Ti. The oxygen reduction reaction (ORR) current, which is related to the amount of H_2_O_2_ generated near Ti^36,37^, increased in the series of Ti-Mg-3wt% Zn < Ti-Mg < Ti-Mg-3wt%Ca, which led to the same conclusion (Fig. 4b). Specifically, for the Ti-Mg-3wt%Ca system, the ORR current at the applied voltage of −0.2 *vs*. Ag/AgCl was −1.3 mA/cm^2^, which was approximately 4.3 and 1.5 times higher than that of the Ti-Mg-3wt% Zn and Ti-Mg systems, respectively. The controllable H_2_O_2_-releasing kinetics of the Ti-Mg alloy system through degradation engineering of Mg alloys indicated its high feasibility with regard to selective bacterial remediation.

### Effects of bi-metal platform on *E.coli* and Zebrafish

**Figure 5:**
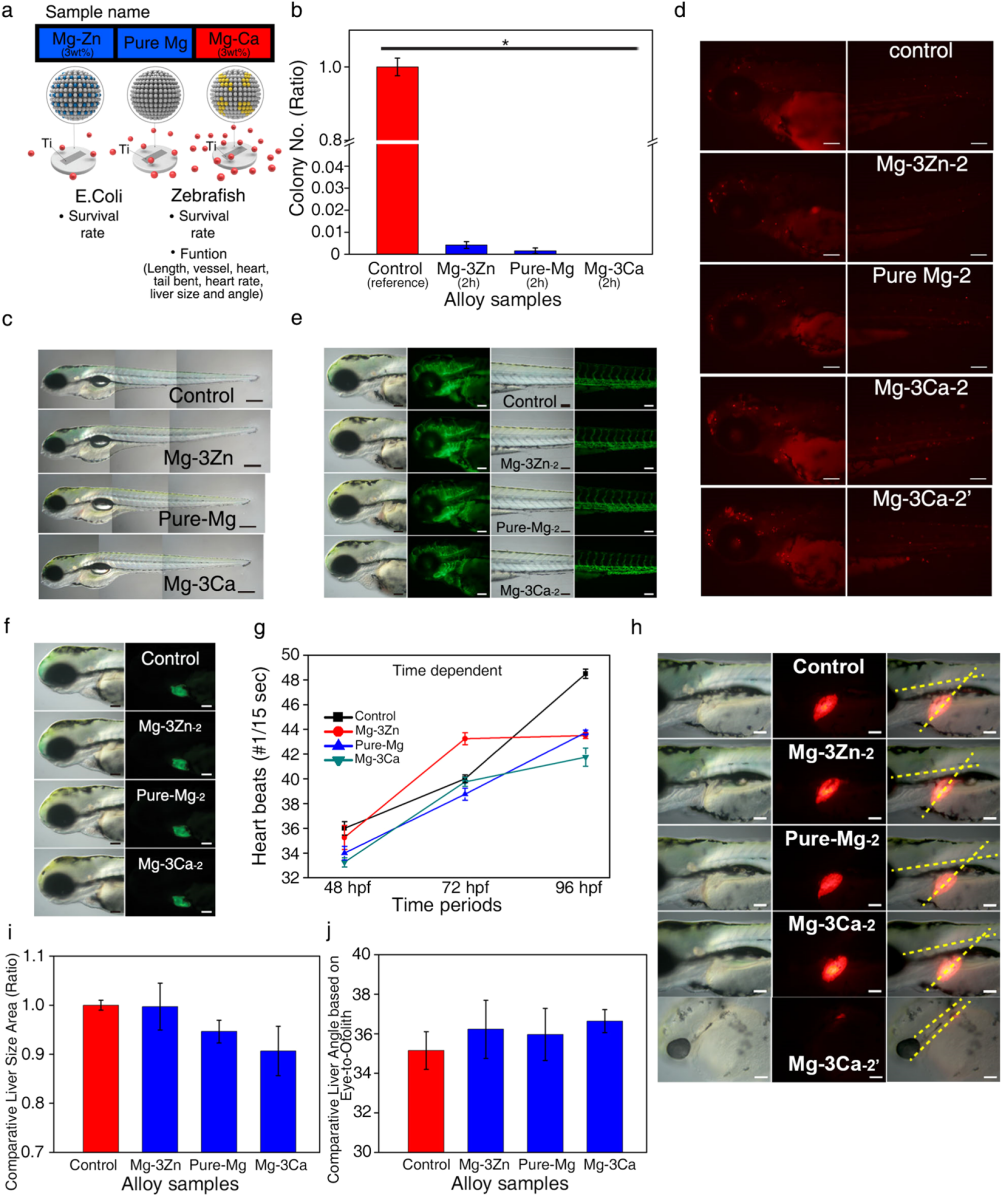
Effect of Ti-Mg-alloy system (Ti-Mg-3wt% Zn, Ti-Mg, and Ti-Mg-3wt%Ca) on the *E. coli* and zebrafish embryos. (**a**) Schematics for the effects of the Ti-Mg alloy systems on the *E. coli* and zebrafish embryos. (**b**) Viability of the *E. coli* treated with the Ti-Mg alloy systems. (**c**) Gross morphology of wild type zebrafish embryos treated with the Ti-Mg alloy systems at 96 hpf from a lateral view. The scale bar represents 200 µm. (**d**) TUNEL assay of wild type zebrafish embryos at 72 hpf treated with the Ti-Mg alloy systems. The scale bar represents 100 µm. (**e,f**) Optical observation of Tg(flk1:EGFP) (**e**) and Tg(cmlc2:EGFP) (**f**) zebrafish embryo phenotypes at 72 hpf and upon treatment with the Ti-Mg alloy systems. The scale bar represents 100 µm.(**g**) Assessment of heart functionality based on zebrafish embryo heart rates following treatment with the Ti-Mg alloy systems at 48 hpf, 72 hpf, and 96 hpf. Results from the three separate experiments are presented as heartbeat numbers compared to the control. Means ± SEM (n = 4). ∗p < 0.01 from the control group. (**h**) Optical observation of Tg(lfabp:DsRed) zebrafish embryo phenotype at 96 hpf upon treatment with the Ti-Mg alloy systems. Angle of developing liver was measured by ImageJ software analysis through the part shown by the dotted line. The scale bar represents 100 µm. (**i,j**) Comparative liver sizes (**i**) and angles (**j**) based on eye-to-otolith in the Tg(lfabp:DsRed) zebrafish embryos treated with the Ti-Mg alloy systems. Results from the three embryo measurements are presented as liver sizes and angles compared to the control. ImageJ (version 1.52a, https://imagej.nih.gov/ij/index.html, Wayne Rasband National Institutes of Health, USA) was used for the quantification of sizes and angles of the liver.

Finally, the antibacterial abilities of the three Ti-Mg alloy systems (Ti-Mg, Ti-Mg-3wt%Ca, and Ti-Mg-3wt% Zn) were evaluated utilizing an *in vivo* assessment platform (Fig. 5a). First, each system was immersed for 2 h in the simulated sea water solution containing *E. coli*, and the system’s antibacterial activity was analyzed by measuring the survival rates of *E. coli*. After 2 h incubation of each system, perfect death of *E. coli* was found in all of the groups (Fig. 5b). This is expected as each system can generate at least 20 μM of H_2_O_2_ after 2 h of treatment (Fig. 4a). In the case of Ti-Mg-3wt% Zn system, degraded Zn, which is also one of well-known antibacterial agents^38^, might also contribute to the complete disinfection of *E. coli* along with H_2_O_2._

After confirming their antibacterial activities, we checked the potential effect of each system on the viability of zebrafish. In the results obtained by phenotypic observation, most of the groups did not induce biological defects (Fig. 5c; Supplementary Table 2); however, when the Ti-Mg-3wt%Ca system was applied to zebrafish embryo-containing solutions, some of the zebrafish embryos near the Ti surface exhibited severe developmental defects (Supplementary Table 2b shadow parts). Similar phenomena were observed in various transgenic zebrafish models (Fig. 5e–j, Supplementary Figs. 6 and 7, and Supplementary Movies 7–14). Incubating the Ti-Mg and Ti-Mg-3wt% Zn groups in the zebrafish-containing solutions for 2 h did not lead to any phenotypic or functional abnormalities in the organ transgenic models (Fig. 5d–j, Supplementary Figs. 6 and 7, and Supplementary Movies 7–14). For example, heart rates, liver sizes, and liver angles of the zebrafish models treated with the Ti-Mg and Ti-Mg-3wt% Zn groups exhibited negligible variations compared to the control group, in which no Ti-Mg alloy system was incorporated (Fig. 5g,i,j and Supplementary Figs. 6 and 7). Only in the case of the Ti-Mg-3wt%Ca group did some zebrafish embryos near the Ti surface show severe liver abnormalities in the observation of the liver transgenic model (Supplementary Table 2b, Fig. 5h bottom panel).

These results were consistent with our previous findings in studies that utilized a standard H_2_O_2_ solution and an *in vivo* assessment platform. In the cases of the Ti-Mg and Ti-Mg-3wt% Zn systems, the amounts of H_2_O_2_ released after 2 h of reaction were 23 μM and 33 μM, respectively, and these values were in the optimum H_2_O_2_ concentration range (from 20 to 50 μM) for selective *E. coli* remediation. However, the Ti-Mg-3wt%Ca system was able to generate 60 μM of H_2_O_2_ at the same reaction time, which was slightly higher than the upper bound of the optimum range, and thus some defects in zebrafish appeared in this group. Moreover, because the H_2_O_2_ formed at the Ti surface, we speculated that the local H_2_O_2_ concentration near the Ti surface was likely higher than that of the bulk solution^39,40^. Consequently, the zebrafish near the Ti surface might have experienced higher oxidative stress and thus suffered severe abnormalities and defects. We also confirmed that the Mg, Zn, and Ca ions released by sacrificial Mg alloys did not induce defects in the zebrafish embryos (Supplementary Table 3), indicating that H_2_O_2_ is the primary factor for these phenomena.

For the long-term application of Ti-Mg alloy system, our future work will be focused on improving the corrosion resistance of Mg through element design and microstructural engineering, as the degradation rate of Mg alloy in our system is faster than that of conventional Mg implant due to the presence of the galvanic circuit between the alloy and Ti. The enhanced corrosion resistance of Mg alloy will also decrease the H_2_O_2_ release kinetics and the side effect of H_2_O_2_ on adjacent cells and tissues. Along with the degradability engineering of Mg alloys, the toxicological evaluation of our system will be more thoroughly evaluated with mammalian models in the future.

## Conclusions

In summary, we propose a new antibacterial technology that utilizes carefully designed Mg alloys, which can harness antibacterial activities when coupled with Ti metal without any surface treatment. By integrating the Ti and Mg alloys, H_2_O_2_, as an oxidizing agent that kills bacteria, is spontaneously generated at the Ti surface through the ORR process. By engineering the microstructural and electrochemical properties of Mg with Ca and Zn alloying elements, we can quantitatively tune the H_2_O_2_ formation kinetics of the Ti-Mg alloy system. In addition, an *in vivo* toxicity assessment platform comprised of *E. coli* and various transgenic zebrafish embryos was constructed in this work to discover the ideal H_2_O_2_ formation kinetics for selective bacterial remediation. Finally, degradability optimization of Mg alloys led to the development of new antibacterial systems, Ti-Mg and Ti-Mg-3wt% Zn, which can selectively remediate *E. coli* without any effects on the survival rate, development, and biological functions of transgenic zebrafish embryos. Based on our preliminary results utilizing metals other than Ti, such as 316 stainless steel, Ni-Ti, and Co-Cr (Supplementary Fig. 8), we envision that this antibacterial strategy using the degradability engineering of Mg alloys could be extended to diverse types of metals.

## Methods

### Growth of bacteria and test of antibacterial activity

Bacterial cultures of *dam*^*−*^*/dcm*^*−*^ *Escherichia coli* (NEB, C2925H) were chosen as test samples, respectively, for the antibacterial activity experiments. The bacteria were grown overnight on LB broth agar (Merck-Millipore, #110285, #110283) on chloramphenicol (Duchefa Biochemie, C0113.0025) plates in an incubator at 37 °C. The resulting bacterial growth was harvested using a sterilized swab and resuspended in 5 ml of simulated sea water solution (Sigma Aldrich, synthetic sea water). This suspension of bacteria was used as a stock suspension for the antibacterial activity tests.

Antibacterial activities were tested in 6-well plates. Bacterial stock suspension (200 μl) was transferred into each well with 3.8 ml of the simulated sea water solution. An aliquot (200 μl) from the bacteria in each well was spread onto the plates of LB broth agar with chloramphenicol. The plates were incubated at 37 °C for 15 hours, and the resulting bacterial growth was counted in terms of colony-forming units (CFU). The bacteria comparative viable ratio was calculated as follows:

$${\rm{Comparative}}\,{\rm{viable}}\,{\rm{ratio}}={\rm{CFUt}}\,({\rm{treatment}}\,{\rm{group}})/{\rm{CFUn}}({\rm{non}}-{\rm{treatment}}\,{\rm{group}}),$$

where the CFUt (treatment group) is the number of colony-forming units measured after plating cells treated in the presence of electrical reactions with electrodes, and CFUn (non-treatment group) is the number of colony forming units measured after plating cells exposed to only the simulated sea water solution. All tests were conducted in triplicate and repeated three times to confirm reproducibility.

### Zebrafish models and growth conditions

This study used the Tg(flk1:EGFP), Tg(cmlc2:EGFP), and Tg(lfabp:DsRed) zebrafish lines, which express green and red fluorescent proteins on the surfaces of blood vessels, cardiac tissue, and liver tissue, and a wild type (standard AB strain) zebrafish. Each zebrafish line was obtained from the Zebrafish Resource Bank (ZOMB) at Kyungpook National University (Daegu, Korea) and maintained at 28 °C under a daily cycle of 14 hours of light exposure and 10 hours of dark conditions^41^. The zebrafish embryos were gathered following the natural mating of their parents. All the experimental methods using the zebrafish models were approved by the Korea Zebrafish Resource Bank (KZRB or ZOMB) of the Kyungpook National University and were performed in accordance with standard proved guidelines and regulations at the zebrafish facility of the Kyungpook National University.

### H_2_O_2_ treatment

H_2_O_2_ (CAS 7722–84–1) was purchased from EMD Millipore (Massachusetts, United States). A stock solution of H_2_O_2_ was prepared in distilled water at a concentration of 20 mM and stored at 4 °C until further use. H_2_O_2_ stock solution was dissolved in zebrafish embryonic water at the experimental concentrations (10, 20, 50, 100, 200, and 500 uM), which were added to 6 well plates. Zebrafish embryos (10 to 20) at 6 hours-post-fertilization (hpf) were treated with the aforementioned experimental concentrations of H_2_O_2_ solutions. ZEISS Stemi 2000, LEICA MZFLIII, ZEISS Imager Z1 and ZEISS axioskop, LEICA S6D, LEICA DMI6000B were used to observe the zebrafish embryos at approximately 72 and 96 hpf. The assay was replicated three times.

### Fabricating Ti-Mg alloy system and investigating the effects of Ti-Mg alloy system on *E. coli* and zebrafish embryos

Pure Mg (99.99 wt%), pure Zn pillet (99.99 wt%) and pure Ca powder (99.99 wt%) were utilized to fabricate cylindrical shaped as-cast Mg alloys (Mg-3wt% Zn, pure Mg, and Mg-3wt% Ca), as described previously^19,29^. In detail, Mg alloys were carefully melted by gravity casting under Ar atmosphere. Then, the molten Mg alloys were transferred into the stainless-steel mold (cylindrical form, 100 mm in diameter and 50 mm in height) over 700 °C. Finally, as-cast Mg alloys were cut into cylindrical form with 11 mm in diameter and 1 mm in height. The chemical compositions of Mg-alloys were measured with inductively coupled plasma analysis (ICP, ARIAN 710-ES).

For fabricating Ti-Mg alloy systems (Ti-Mg-3wt% Zn, Ti-Mg, and Ti-Mg-3wt% Ca), pure Ti bar (20 mm × 10 mm × 50 mm) were firstly fixed at the bottom of the abovementioned stainless-steel mold. Then, molten Mg alloys were poured into the mold containing the Ti bar. Finally, as-cast Ti-Mg alloys were cut into the cylindrical form with 11 mm in diameter and 1 mm in height.

To investigate the effects of fabricated Ti-Mg alloy systems on *E. coli*, Ti-Mg alloys were immersed in 3.8 ml of the simulated sea water solution containing 200 μl of the bacterial stock suspension for 2 hours and then the bacteria comparative viable ratio was calculated. Similarly, 10 to 20 zebrafish embryos at 6 hours-post-fertilization (hpf) in zebrafish embryonic water were treated with Ti-Mg alloys for 2 hours to examine their effects on zebrafish embryos. ZEISS Stemi 2000, LEICA MZFLIII, ZEISS Imager Z1 and ZEISS axioskop, LEICA S6D, LEICA DMI6000B were used to examine zebrafish embryos at approximately 72 and 96 hpf. The assay was replicated three times.

### Examination of zebrafish heart functionality

For the control group, the heartbeat counts of wild-type zebrafish at 48, 72, and 96 hbf were measured, as described previously^21^. In the case of experimental groups where zebrafish embryos were treated with H_2_O_2_ solution or Ti-Mg alloy system at 6 hbf, we measured the heartbeat counts of treated zebrafish at 48, 72, and 96 hpf. Four zebrafish was utilized for each experimental condition to measure the number of heartbeats within 15 s.

### Apoptotic cell detection in zebrafish embryos (TUNEL assay)

DNA fragmentation was detected using the *In Situ* Cell Death Detection Kit, TMR red (Cat. No. 12156792910 Roche, Sigma, St. Louis, United States) according to the manufacturer’s protocol^42,43^. Embryos at 72 hpf were treated with solution, and alloys were fixed overnight at 4 °C in a 4% paraformaldehyde solution. Fixed embryos were washed three times with PBS and then were incubated in a permeabilization solution for 1 hour at room temperature. After washing with PBS, embryos were treated with TUNEL solution mixtures for 60 minutes at room temperature in the dark and then washed with PBS 3 times before optical observation.

### Analysis of the liver size and angle

Developmental liver size and angle were analyzed in the Tg(lfabp:DsRed) zebrafish embryos. We observed the liver at 96 hpf for the control group and experimental groups of zebrafish. Images from the experimental groups were processed *via* ImageJ (version 1.52a, https://imagej.nih.gov/ij/index.html, Wayne Rasband National Institutes of Health, USA) for the quantification of sizes and angles of fluorescent parts.

### Statistical analysis

One-way ANOVA and Student’s t-test were performed to assess the significance of the differences among the experimental groups. The level of significance was set at p < 0.01. The results are represented as means ± SEM (standard error of the mean).

### Electrochemical analysis

Electrochemical evaluations were performed by utilizing a one compartment electrochemical cell with a conventional three-electrode system. Simulated sea water solution were added into the electrochemical cell and utilized as the electrolyte. For CV analyses, Ti plate, Mg-alloy plate, and Ag/AgCl reference electrode (BASi, Ag/AgCl/3 M NaCl) were used as working, counter, and reference electrodes, respectively. The CV curves were recorded from 0.3 V to −0.5 V (*vs*. Ag/AgCl) in O_2_- or N_2_-saturated electrolytes at a scan rate of 5 mV/s. For OCP measurements, Mg-alloy plate, platinum plate, and Ag/AgCl reference electrode (BASi, Ag/AgCl/3 M NaCl) were utilized as working, counter, and reference electrodes, respectively. All of the measurements were conducted at 37 ± 0.5 °C with a potentiostat (CHI 760 C, CH Instruments, Inc., USA).

### H_2_O_2_ Spectroscopy measurement

The amount of H_2_O_2_ generated from the Ti-Mg alloy system was determined by using a fluorometric hydrogen peroxide assay kit (Sigma-Aldrich, USA). According to the manufacturer’s protocol, after each system had been immersed in the simulated sea water solution for a specified amount of time, the solution was then collected and mixed with hydrogen peroxide assay buffer. Next, the fluorescence intensity (λ_ex_ = 540 nm, λ_em_ = 590 nm) of the mixed solution was measured using a fluorescence plate reader (Infinite F200 Pro, Tecan, Switzerland) to estimate the amount of H_2_O_2_ released in each solution.

### Mg alloys characterizations

The SEM images of the Mg alloys were obtained using Quanta 3D PEG (FEI, Netherland) with 5 keV of electron beam energy and 11.8 pA of electron current. Samples were not sputter-coated for the SEM analyses. To obtain SEM energy dispersive X-ray spectroscopy (EDS) spectra, electrons were accelerated to 15 keV of beam energy. X-ray diffraction (XRD) patterns of Mg alloys were acquired using a Bruker D-8 Advance X-ray diffractometer with Cu Kα radiation (λ = 1.54056 Å) and a scan angle from 5° to 80° with a step size of 0.02°.

### Immersion test

Immersion tests carried out in the simulated sea water solution at 37 ± 0.5 °C. Each Mg alloy plate was suspended in the solution, and funnels were placed over the specimens to collect the evolved hydrogen gas. The volume of hydrogen produced was measured over time.

### Work functions of pure Mg, Mg_2_Ca and Mg-3.3 wt% Zn alloy

We performed the first principle calculations to obtain the work functions of pure Mg, Mg_2_Ca and Mg-3.3 wt% Zn solid solution. Density functional theory (DFT) calculations using the VASP program packages were used^44^. The plane wave basis expansions with an energy cutoff of 300 eV and the generalized gradient approximation (GGA) with the PW91 exchange-correlation functional were used. The core-valence interaction is described by the projector-augmented wave (PAW) method^45^. We constructed pure Mg, Mg_2_Ca and Mg-3.3 wt% Zn slab structure to calculate work function as shown in Supplementary Fig. 3. Vacuum sizes are given larger than 20 Å to minimize interaction between slabs. Supplementary Fig. 4 shows the calculated work functions of pure Mg, Mg_2_Ca and Mg-3.3 wt% Zn solid solution. The work function decreases in the series of Mg-3.3 wt% Zn > pure Mg > Mg_2_Ca, which shows good agreement with the variation of OCP as shown in Fig. 3e of the manuscript.

## Supplementary information

Supplementary Information.

Supplementary Movie 1.

Supplementary Movie 2.

Supplementary Movie 3.

Supplementary Movie 4.

Supplementary Movie 5.

Supplementary Movie 6.

Supplementary Movie 7.

Supplementary Movie 8.

Supplementary Movie 9.

Supplementary Movie 10.

Supplementary Movie 11.

Supplementary Movie 12.

Supplementary Movie 13.

Supplementary Movie 14.
