# Supplementary material for: Tailoring H2O2 generation kinetics with magnesium alloys for efficient disinfection on titanium surface
Source: Sci Rep. 2020 Apr 16;10:6536. doi: 10.1038/s41598-020-63007-6 (PMC7162939; doi:10.1038/s41598-020-63007-6)
Supplement: Supplementary file 1 — Supplementary Information. [file 41598_2020_63007_MOESM1_ESM.docx]

**Tailoring H_2_O_2_ generation kinetics with magnesium alloys for efficient disinfection on titanium surface**

**Authors:** Jimin Park^1,2†^, Gun Hyuk Jang^2,3,4†^, Yeon Wook Jung^2,5†^, Hyunseon Seo^2^, Hyung-Seop Han^2^, Joonho Lee^5^, Youngmin Seo^2^, Hojeong Jeon^2,3^, Myoung-Ryul Ok^2^, Pil-Ryung Cha^6^, Hyun-Kwang Seok^2,3^, Kwan Hyi Lee ^2,3*^ and Yu-Chan Kim^2,3*^

**Affiliations:**

^1^Department of Materials Science and Engineering, Massachusetts Institute of Technology, Cambridge, Massachusetts, 02139, USA.

^2^Center for Biomaterials, Korea Institute of Science & Technology, Seoul 02792, Republic of Korea

^3^Division of Bio-Medical Science and Technology, KIST School, Korea University of Science and Technology, Seoul 02792, Republic of Korea

^4^Research & Development, NuclixBio, Seoul 08380, Republic of Korea

^5^Department of Materials Science and Engineering, Korea University, Seoul 02481, Republic of Korea

^6^School of Advanced Materials Engineering, Kookmin University, Seoul, 02707, Republic of Korea

^†^ **These authors contributed equally to this work**

***Correspondence and requests for materials should be addressed to:**

Kwan Hyi Lee, Ph.D. (kwanhyi@kist.re.kr)

Yu-Chan Kim, Ph.D. (chany@kist.re.kr)

**
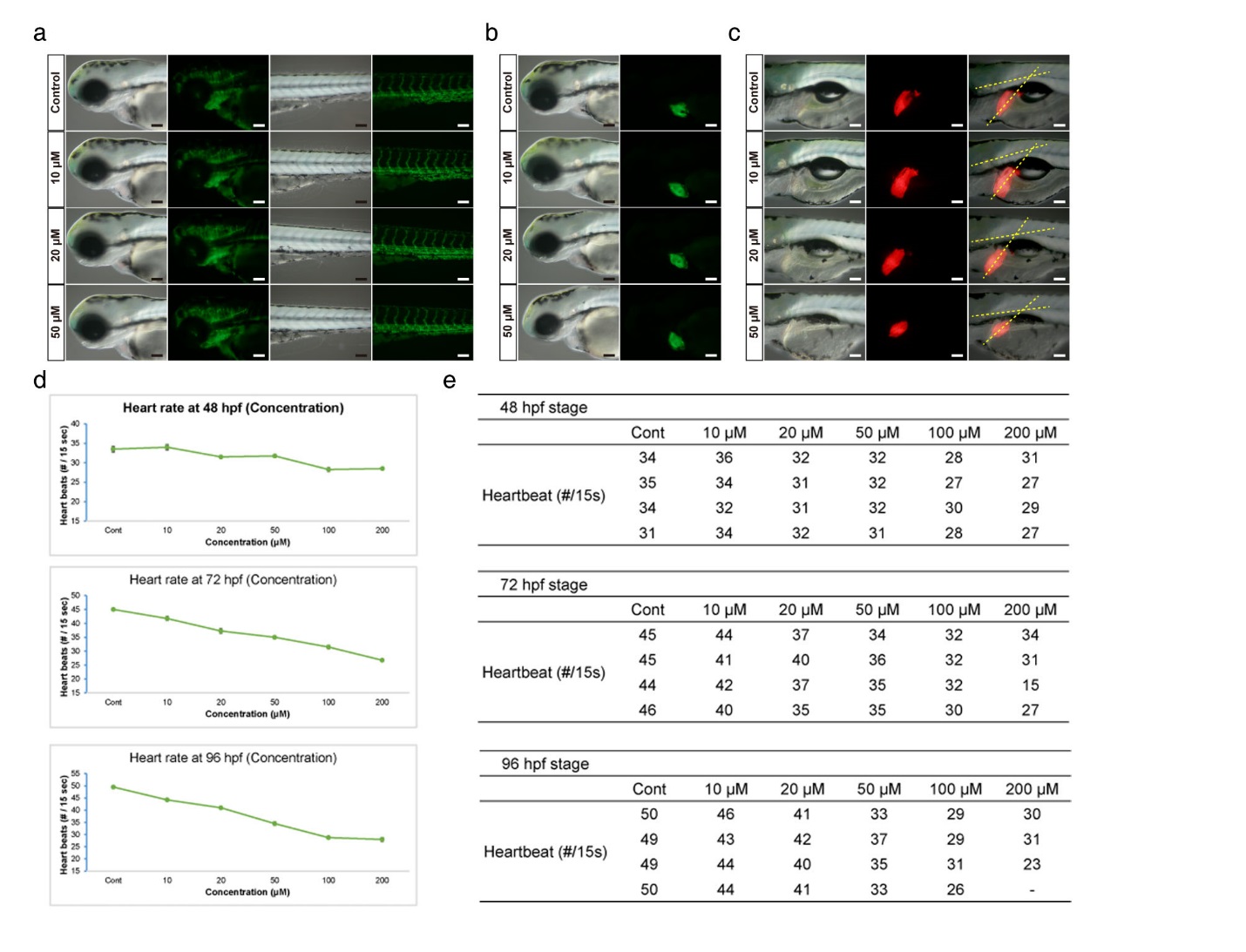
**

Supplementary Figure 1. Optical observation of transgenic zebrafish embryos upon treatment with hydrogen peroxide (48, 72, and 96 hpf). **a, b** Observations of Tg(flk1:EGFP) (**a**) and Tg(cmlc2:EGFP) (**b**) zebrafish embryo phenotypes at 72 hpf upon treatment with H_2_O_2_. **c** Tg(lfabp:DsRed) zebrafish embryo phenotype at 96 hpf upon treatment with H_2_O_2_. **d** Assessment of heart functionality based on zebrafish embryo heart rates following treatment with H_2_O_2_ at 48 hpf, 72 hpf, and 96 hpf (control, 10, 20, and 50 uM). **e** Heart rate numbers of zebrafish embryos treated with various concentrations of the H_2_O_2_. The scale bar represents 100 µm.


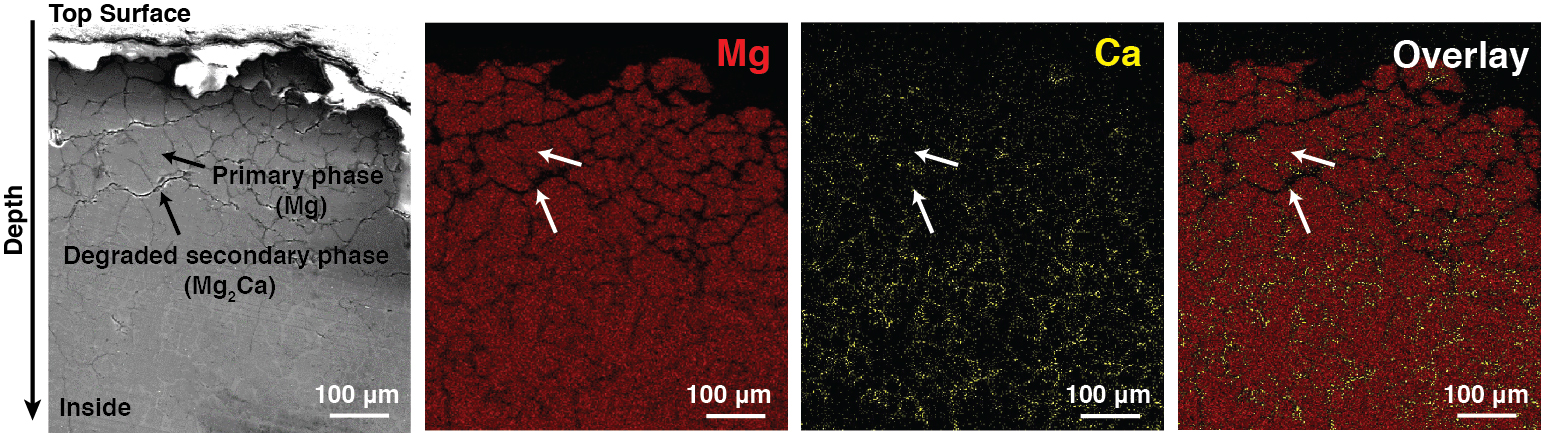


**Supplementary Figure 2.** Cross-sectional SEM images and corresponding EDX spectrum of Mg-3wt%Ca sample after 2 weeks of degradation. Only Mg signals appeared in the non-degraded regions near the top surface, indicating that this region is primary Mg phase. The microstructure of degraded region near the top surface was similar to that of Mg_2_Ca secondary phase in the as-cast Mg-3wt%Ca alloy sample, implying that the degraded region was mainly comprised of Mg_2_Ca phase. Ca signals were detected from the regions inside the sample with microstructure similar to that of degraded region near the top surface.


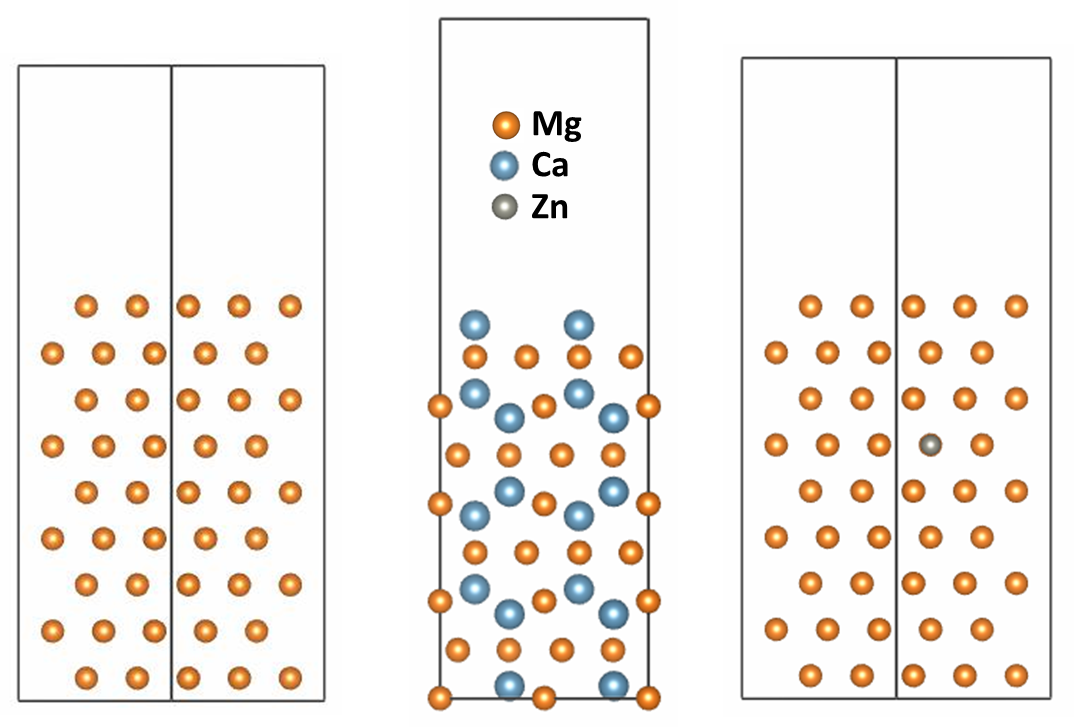


Supplementary Figure 3. Atomic configurations of pure Mg (left), Mg_2_Ca (center) and Mg-3.3wt%Zn (right) solid solution used in the work function calculation. The slab structures are composed of 81 Mg atoms for pure Mg, 32 Mg and 16 Ca atoms for Mg_2_Ca and 80 Mg and 1 Zn atoms for Mg-3.3wt%Zn.

Supplementary Figure 4. Calculated work functions of pure Mg, M_g2_Ca and Mg-3wt%Zn solid solution.


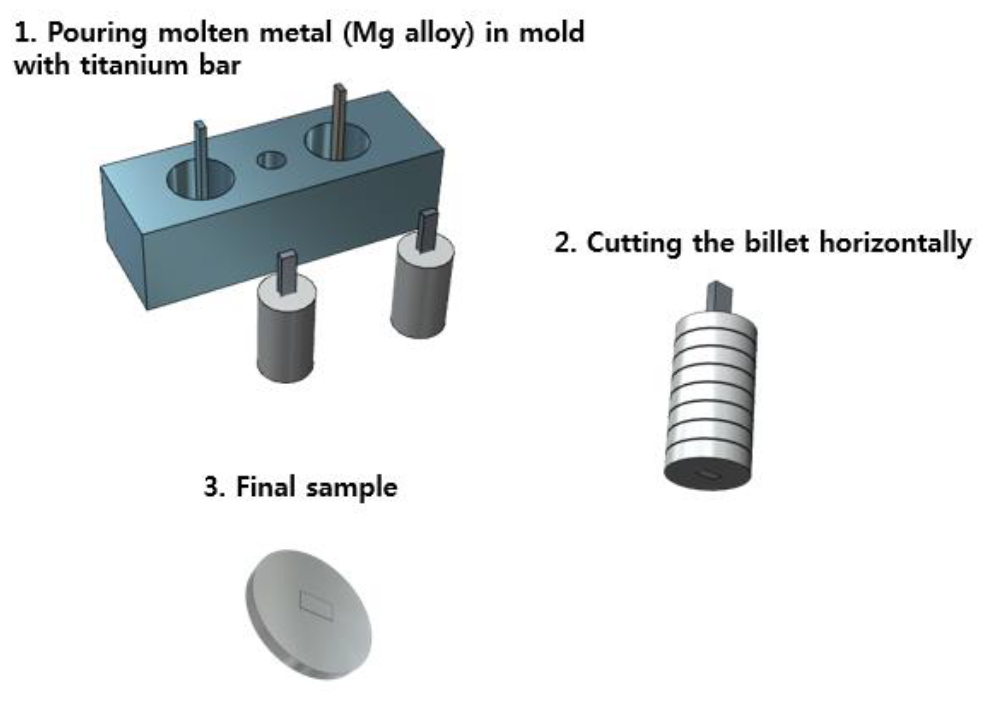


Supplementary Figure 5. Fabricating process of Ti-Mg alloy systems.

**
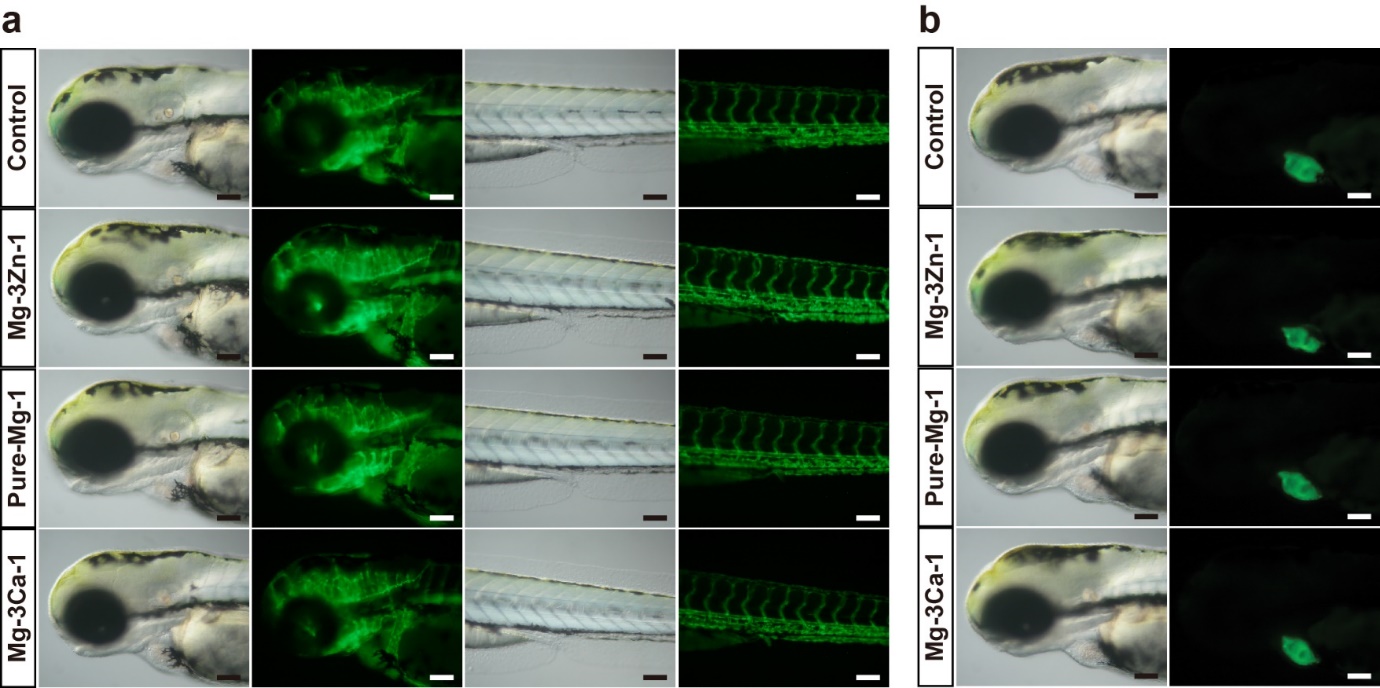
**

Supplementary Figure 6. Optical observation of Tg(flk1:EGFP) and Tg(cmlc2:EGFP) zebrafish embryos following Ti-Mg alloy pretreatment (72 hpf). **a, b** Observation of Tg(flk1:EGFP) (**a**) and Tg(cmlc2:EGFP) (**b**) zebrafish embryo phenotypes at 72 hpf. The scale bar represents 100 µm.

**
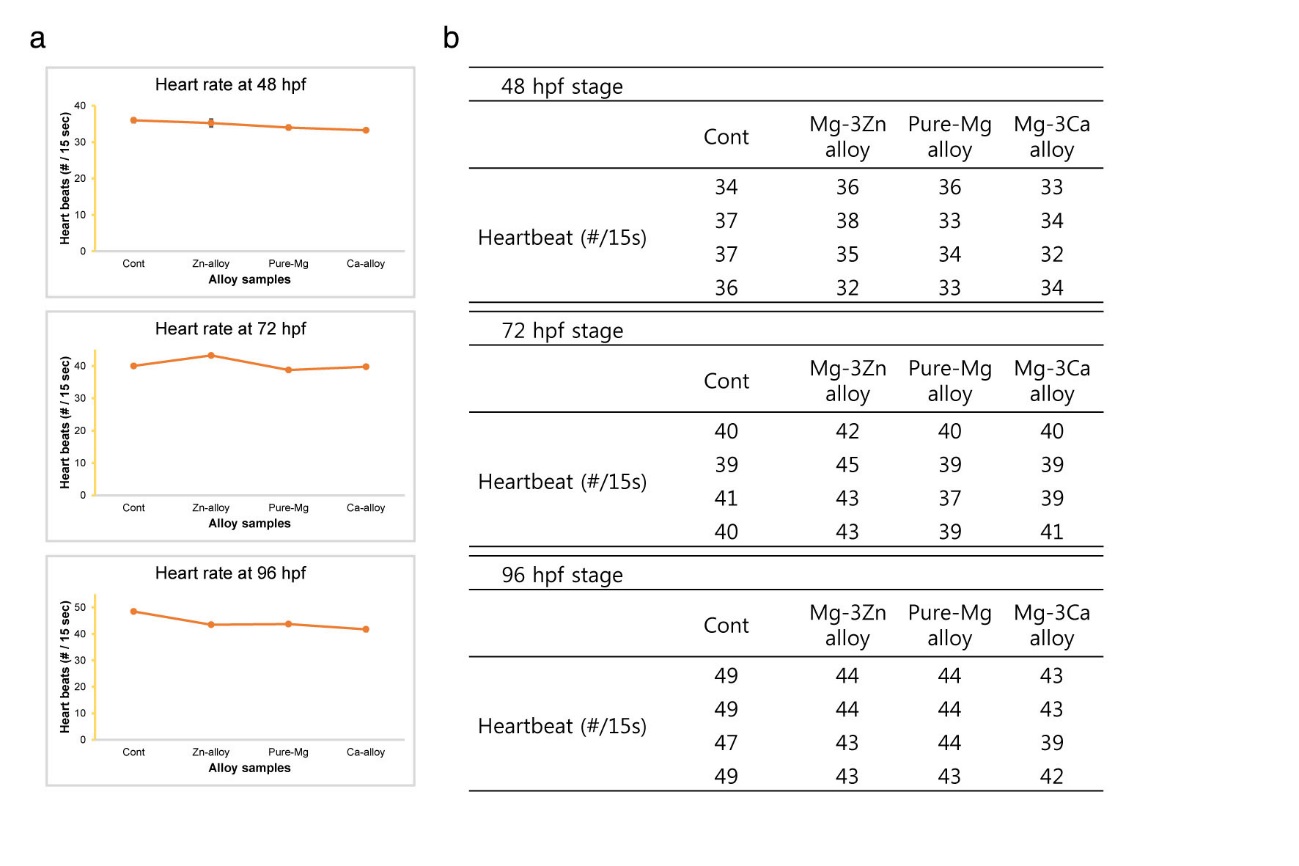
**

Supplementary Figure 7. Observation of Tg(cmlc2:EGFP) zebrafish embryo upon simultaneous treatment with the Ti-Mg alloy systems. **a** Assessment of heart functionality based on zebrafish embryo heart rates following treatment with the Ti-Mg alloy systems. **b** Heart rate numbers of zebrafish embryos treated with the Ti-Mg alloy systems.

**
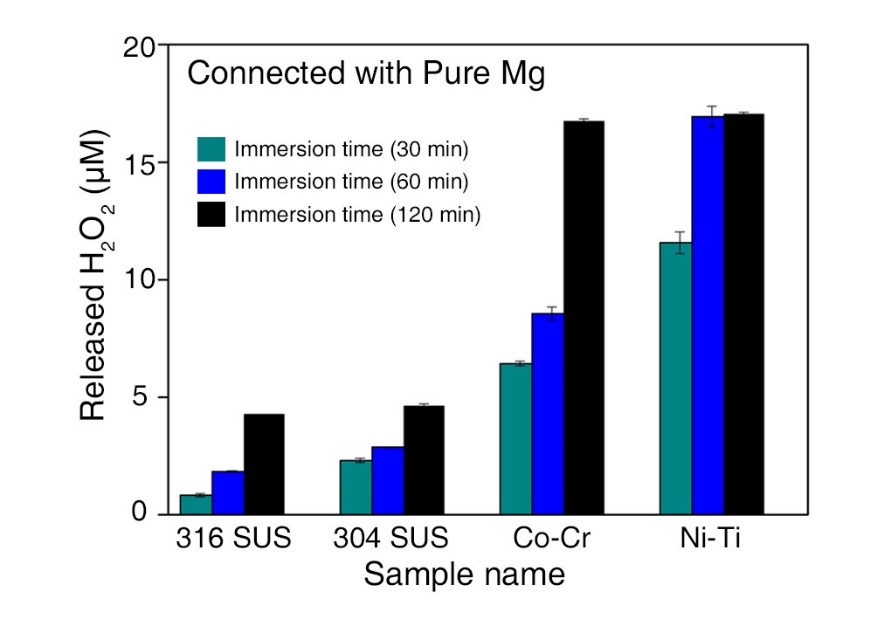
**

Supplementary Figure 8. H_2_O_2_ releasing behaviors in other metals-Mg alloys system.

**
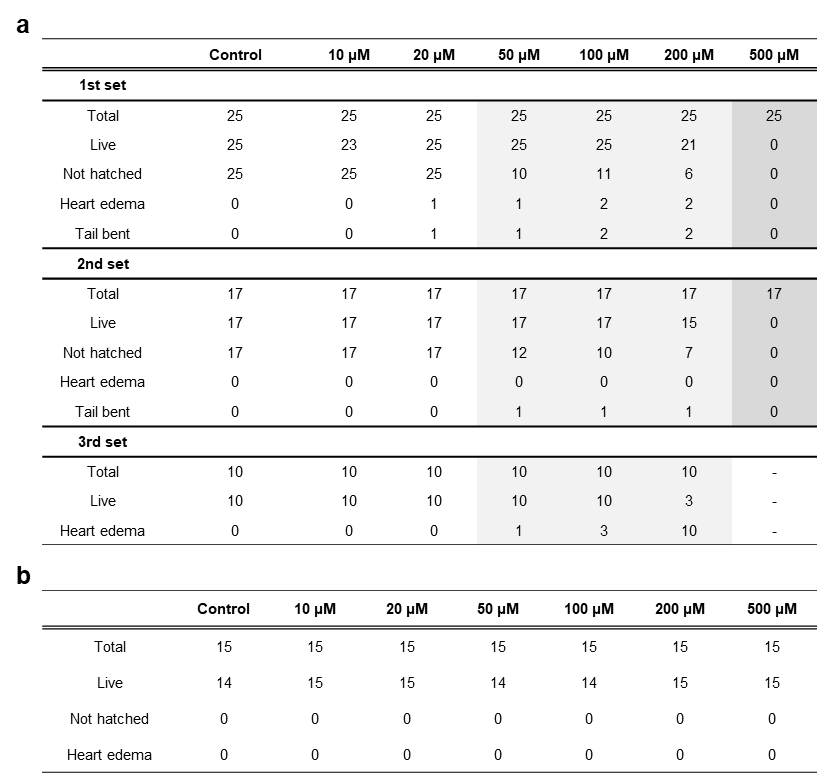
**

Supplementary Table 1. Quantification of the phenotypic differences among zebrafish embryos upon treatment with various concentrations of hydrogen peroxide. **a, b** Quantification of phenotypic differences upon continuous treatment with H_2_O_2_ (**a**) and during 2 hours (**b**) in the 6 hpf stage.

**
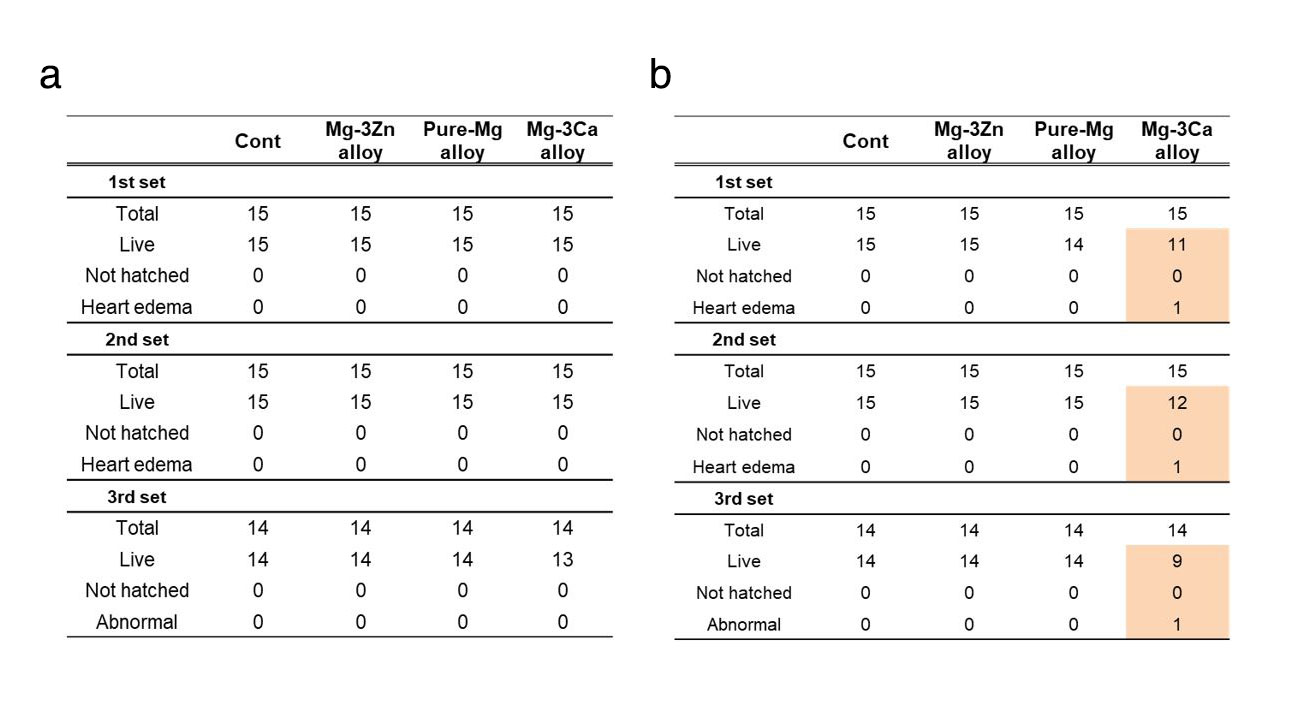
**

Supplementary Table 2. Quantification of the phenotypic differences among zebrafish embryos upon treatment with the Ti-Mg alloy systems. **a, b** Groups of treated embryos with preferential treatment and removal of alloys (**a**) and simultaneously treated alloys and zebrafish embryos (**b**).

**
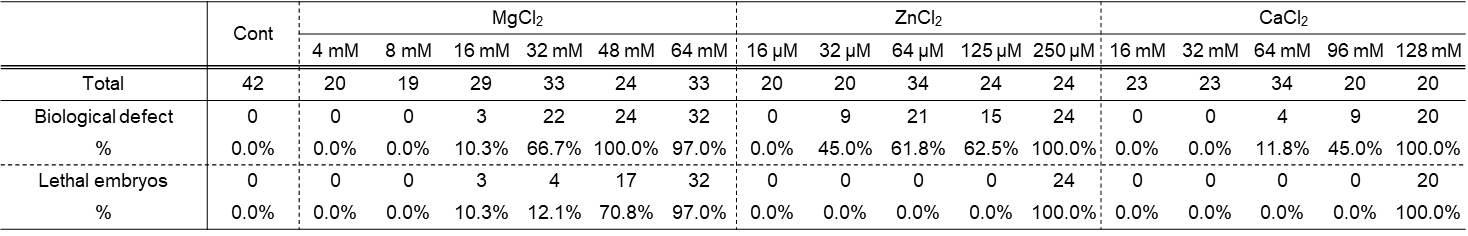
**

Supplementary Table 3. Quantification of the biological defects and mortality rates of zebrafish embryos upon treatment with various concentrations of metal ions (Zn, Mg, Ca).

Supplementary Movie 1 to 6. Beating heart of the control Tg(cmlc2:EGFP) zebrafish embryo at 72 hpf and Tg(cmlc2:EGFP) zebrafish embryo at 72 hpf treated with H_2_O_2_ (10, 20, 50, 100, and 200 μM).

Supplementary Movie 7 to 10. Beating heart of the control Tg(cmlc2:EGFP) zebrafish embryo at 72 hpf and Tg(cmlc2:EGFP) zebrafish embryo at 72 hpf upon incubation with the Ti-Mg alloy systems (Mg-3wt%Zn, pure-Mg, and Mg-3wt%Ca).

Supplementary Movie 11 to 14. Beating heart of the control Tg(cmlc2:EGFP) zebrafish embryo at 72 hpf and Tg(cmlc2:EGFP) zebrafish embryo at 72 hpf upon simultaneous treatment with the Ti-Mg alloy systems (Ti-Mg-3wt%Zn, Ti-Mg, and Ti-Mg-3wt%Ca).
